# Accelerometer-measured physical activity patterns are associated with phenotypic age: Isotemporal substitution effects

**DOI:** 10.1016/j.heliyon.2023.e19158

**Published:** 2023-08-23

**Authors:** Yanwei You, Yuquan Chen, Xiaoxin Wang, Mengxian Wei, Qi Zhang, Qiang Cao

**Affiliations:** a Division of Sports Science & Physical Education, Tsinghua University, Beijing 100084, China; b Institute of Medical Information/Medical Library, Chinese Academy of Medical Sciences & Peking Union Medical College, Beijing 100020, China; c Department of Nutrition and Food Hygiene, Public Health College, Harbin Medical University, Harbin 150081, China; d Undergraduate Department, Taishan University, Taian 250111, China; e Department of Earth Sciences, Kunming University of Science and Technology, Kunming 650093, China; f School of Pharmacy, Macau University of Science and Technology, Macau 999078, China

**Keywords:** Physical exercise, Sedentary behavior, Phenotypic age, Isotemporal substitution model, Cross-sectional study

## Abstract

Prolonged sitting appears to accelerate aging, while optimal physical activity patterns have been found to delay the process. It is an emerging topic, and no conclusions have been reached regarding the relationship between physical activity patterns and biomarkers-measured aging. Hence, the aim of this study was to investigate the association between sensor-based objectively measured physical activity and phenotypic age using a nationwide population from the National Health and Nutrition Examination Survey (NHANES) in the United States. Weighted linear regression models were performed to evaluate the association between sedentary behavior, light-intensity physical activity (LPA), moderate-to-vigorous physical activity (MVPA) and phenotypic age. A total of 6439 eligible participants were included and the weighted respondents were 49,964,300. Results showed that prolonged sitting was positively associated with phenotypic age in the fully adjusted model [β (95% CI): 0.009(0.007,0.011), p < 0.001], while increasing volume of LPA and MVPA was associated with younger phenotypic age using the fully adjusted model [β (95% CI): −0.010(-0.013,-0.006), p < 0.001; −0.062(-0.075,-0.048), p < 0.001]. By utilizing the Isotemporal Substitution Model, it was found that replacing 30 min of sedentary behavior with 30 min of LPA or MVPA per day was associated with estimated 0.4 or 1.9 years of phenotypic age reduction. According to the study's findings, maintaining a certain level of physical activity could delay the process of aging and intensity matters.

## 1 Introduction

Aging is a physiological process that is associated with health issues including changes in body composition and biochemistry [1,2]. Biomarkers are indicators of these changes and can be a determinant factor in understanding the progress of changes associated with aging. Empirical evidence has elucidated that the process of aging manifests in a multitude of biomarkers, encompassing a diverse range of physiological and immunological factors [3,4]. Biomarkers of aging are measurable characteristics or substances that provide information about the biological aging process [5]. Taking telomere length as an example, telomere length (the protective caps on chromosomes that shorten as individuals get older) has been found to be an easy-assessable indicator of aging [6,7]. In brief, aging-related chromosome shortening, specifically telomere shortening, is problematic because it is associated with cellular dysfunction, genomic instability, and increased vulnerability to age-related diseases. However, using a single biomarker could not adequately capture the complexity of the aging trajectory. It is necessary to develop a novel index to better evaluate the aging process.

As a classic index, chronological age is simply a measure of time since birth, which has been widely used to reflect the aging process. However, it does not consider physiological, lifestyle and environmental factors. Phenotypic age, as an emerging index refers to the biological age of an individual, is determined by measuring multiple physiological and biological changes associated with aging. The specific phenotypic age in this study used chronological age and 9 biomarkers with reference to previous studies [[8], [9], [10]]. According to Morgan E. Levine [8], phenotypic aging approaches in combination with biological and clinical markers could effectively predict mortality risks, cause-specific mortality risks, cognitive declines, and facial aging risks [[11], [12], [13]]. Consequently, novel phenotypic age seems to be a reliable index that assesses individual health in a more comprehensive manner.

It has been shown that engaging in regular physical activity has numerous beneficial effects on the aging process. These include a delayed onset of several age-related diseases involved in aging, such as cardiovascular disease, cerebrovascular accidents, type 2 diabetes, and certain types of cancer [[14], [15], [16]]. Physical exercise is a subset of physical activity, and more specifically, recreational or leisure-time physical activity is often referred to as exercise. Exercise (especially balance and functional exercises) has been shown to be beneficial in maintaining and improving physical functions, which might be beneficial for fall prevention [17]. Additionally, physical exercise is linked to enhanced cognitive function and amplified mental health [18,19], including improved working memory, diminished rates of anxiety and depression. In contrast, sedentary behavior, defined as prolonged periods of sitting or inactivity, has been associated with a range of deleterious health outcomes and an increased risk of age-related diseases [20,21]. Furthermore, a previous study reported that a higher 24-h percentile for total movement activity was associated with better cognitive health in elderly groups [22].

There are two main methods of measuring physical activity patterns: a subjective questionnaire and an objective accelerometer. Self-reported measures, such as questionnaires and diaries, are subjective and rely on an individual's recall and interpretation of their physical activity [23]. These measures are susceptible to bias and error, and can result in under- or over-reporting of physical activity levels [24]. On the other hand, accelerometers, for example, are device-measurement of physical activity. Accelerometers use sensors to directly measure movement and provide a more accurate assessment of physical activity patterns, which have been identified in several previous studies [18,25,26]. For these reasons, accelerometers can partially reduce measurement error by questionnaires and improve study findings accuracy. To identify the effects of different physical activity patterns on health outcomes, the Isotemporal Substitution Model is an emerging statistical approach that uses regression analysis to estimate the influence of replacing time spent in one physical activity with time spent in another [27]. However, the association between different physical activity patterns (especially sedentary behavior and sensor-measured physical activity) and biomarker-based phenotypic age remains largely unknown.

To better quantitatively investigate the association between physical activity behavior and the aging process, it was necessary to explore the association between physical activity patterns and phenotypic age, as well as the time-substitution effect. Thus, using a nationwide-based population from the National Health and Nutrition Examination Survey (NHANES), this research aimed to (i) investigate the relationship between physical activity patterns and phenotypic age; (ii) utilize the Isotemporal Substitution Model to explore the substitute effect of physical exercise and sedentary behavior on phenotypic age. We hypothesized that individuals who were more active tended to have a younger phenotypic age than those who were less active.

## 2 Methods

### 2.1 Study participants

This study was a cross-sectional study using data from the National Health and Nutrition Examination Survey (NHANES), an annual nationwide sample survey designed for collecting data on the civilian population, mainly focusing on the health and nutritional status of adults and children in the United States. This program was conducted since 1999 to provide information on approximately 10,000 non-institutionalized household Americans over a two-year period through a multistage probability sampling design. To provide estimates representative of the non-citizen US population, sampling weights were used to account for unequal probabilities of selection and non-responses. Moreover, NHANES was conducted by the National Center for Health Statistics, an agency of the Centers for Disease Control and Prevention (CDC).

**Fig. 1:**
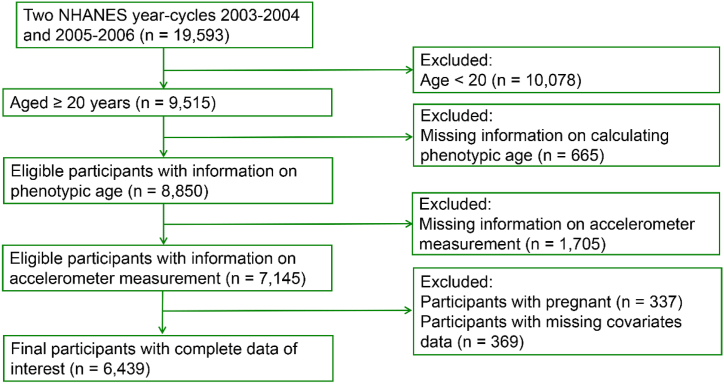
Flowchart depicting the inclusion and exclusion criteria.

Based on two continuous cycles of NHANES 2003/2004 and 2005/2006 (sensor-based physical activity data was available on these two year cycles), a total of 19,593 participants were initially included. 9515 participants were left after excluding those younger than 20 years (n = 10,078). The reason to include participants aged 20 and above was due to the fact that several biomarkers used for calculating phenotypic age were not tested in groups younger than 20 years. Subsequently, we excluded participants with missing biomarker information for calculating phenotypic age (n = 665). Following that, participants with no accelerometer measurement data (n = 1705) were excluded. Furthermore, participants who were pregnant (n = 337) or had missing covariate data (n = 369) were excluded. The final analysis included 6439 participants (Fig. 1).

### 2.2 Measurement of phenotypic age

It comes to the forefront that designing a novel phenotypic age rather than using the independent chronological age predicts better in the heath related outcomes. Referring to the definition of phenotypic age proposed by Morgan E. Levine et al. [8], we calculated the phenotypic age using ten aging related variables, including chronological age, albumin (liver), creatinine (kidney), glucose (metabolic), C-reactive protein (inflammation), lymphocyte percent (immune), mean cell volume (immune), red blood cell distribution width (immune), alkaline phosphatase (liver), and white blood cell count (immune). Blood samples were taken at the mobile examination center. These samples were collected in a standard way and stored in a secure facility. More information about blood samples collecting and processing was reported elsewhere [28,29]. The calculation method for phenotypic age was as follows:

[1] $$Phenotypic\;age=141.50+\frac{\mathrm{Ln}\left[-0.00553\times \mathrm{Ln}\left(\exp\left(\frac{-1.51714\times \exp\left(xb\right)}{0.0076927}\right)\right)\right]}{0.09165}$$

In the formula [1], the parameter xb = −19.907 + 0.0804 × chronological age − 0.0336 × albumin +0.0095 × creatinine + 0.1953 × glucose + 0.0954 × Ln (C-reactive protein) − 0.0120 × lymphocyte percent + 0.0268 × mean cell volume + 0.3306 × red cell distribution width + 0.00188 × alkaline phosphatase + 0.0554 × white blood cell count. The algorithm and detailed calculation methods have been described in the previous literature [[8], [9], [10]].

### 2.3 Measurement of physical activity patterns

Physical activity patterns were characterized as the percentages of time spent in the three daily physical activity behaviors: sedentary behavior, light-intensity physical activity (LPA), and moderate-to-vigorous physical activity (MVPA). With a sensor-based ActiGraph AM-7164 accelerometer (ActiGraph, LLC, Fort Walton Beach, Florida, USA), sedentary behavior, LPA, and MVPA were measured objectively following the protocol outlined previously [30,31]. Accelerometer data for any subject was recorded only once a week, either in 2003–2004 or 2005–2006. This data in NHANES was collected via a device worn on the waist for seven consecutive days. Participants were asked to wear the belt except when sleeping or bathing. The device records the amount of physical activity and sedentary behavior over a seven-day period. Counts of accelerations were integrated over 1-min epochs and analyzed according to CDC quality assurance guidelines [30,32]. In the uniaxial Actigraph, vertical acceleration is measured and recorded as 'counts', indicating the intensity of locomotion-related physical activity. The accelerometer data were recorded for one week in 1-min epochs. A daily average of the time spent on each behavior was calculated over all available valid days. We included participants who had a minimum of 1 valid day of physical activity data. For a day of physical activity data to be considered valid, participants were required to have at least 10 h of valid wear time data. Any day with less than 10 h (600 min) of wear time data was not considered as a valid day and was excluded from the analysis. Referring to previous settings, we set standard count per minute (CPM) thresholds on the basis of the classification of each 1-min epoch: sedentary behavior (<100 CPM), LPA (100–2020 CPM) or MVPA (>2020 CPM) [33]. Units for sedentary behavior, LPA, and MVPA were assessed as minutes per day (minutes/day).

### 2.4 Covariate assessment

Referring to previous settings of analyzing NHANES data [[34], [35], [36]], the twelve variables that were considered to be covariates in the analysis of the effect of physical activity on phenotypic age included age, sex, race, marital status, education (below high school, high school, and college or above), poverty status (low income (<1), middle income [1,3], and high income (>3)), body mass index (BMI), smokers, alcohol drinkers, hypertension, cardiovascular disease (CVD), and diabetes mellitus (DM). To assess smoking and alcohol consumption, questionnaires were used, and smoking conditions were categorized as never, former, and current. Alcohol users were divided into three categories: nondrinker, moderate alcohol use, and heavy alcohol use.

Additionally, we evaluated individuals' chronic diseases. In the present study, hypertension was diagnosed among participants whose systolic blood pressure and diastolic blood pressure exceeded 140 mmHg and 90 mmHg, respectively. CVD was determined by the self-reported presence of congestive heart failure, coronary heart disease, angina, heart attack, or stroke. DM was identified according to the following criteria: (1) a doctor's diagnosis; (2) glycohemoglobin HbA1c (%) > 6.5; (3) fasting glucose (mmol/l) ≥ 7.0; (4) random blood glucose (mmol/l) ≥ 11.1; (5) 2-h Oral Glucose Tolerance Test (OGTT) blood glucose (mmol/l) ≥ 11.1; and (6) use of diabetes medication or insulin. Further details concerning the covariates utilized in this study can be obtained from the National Center for Health Statistics (NCHS) website at http://www.cdc.gov/nchs/nhanes/.

### 2.5 Statistical analyses

Using the NHANES protocol, all data were integrated into one dataset and a weighting methodology was applied based on masked variance. To address nonresponse, noncoverage, and unequal probabilities of selection, weights from Mobile Examination Center (MEC) interviews were reweighted to combine 4 years' worth of NHANES 2003–2006 survey data. All analyses were conducted using R (version 4.1.2, R Core Team) and R Studio (version 2022.07.1 Build 554, R Studio, PBC, Boston, MA) as the programming environment. The complex multistage sampling design and survey weighted analytical methods were applied according to the guidance of the NHANES website, which can be found here: https://wwwn.cdc.gov/nchs/nhanes/analyticguidelines.aspx. The complex multistage sampling statistical methods included weighting, clustering, and stratification to adjust for the probability of selection and provided nationally representative estimates. The weighted procedure was calculated using the “survey” package in R software. This package was designed to analyze complex survey samples, taking into account survey weights. Statistical significance was determined by a p-value of less than 0.05 in all analyses.

In the present study, physical activity patterns [sedentary behavior, low-intensity physical activity (LPA), and moderate-to-vigorous physical activity (MVPA)] were initially analyzed as continuous variables and then re-analyzed as categorized variables, classified into four groups according to quantile points. Participants with missing values were excluded from the main analyses. To examine the association between exposure (sedentary behavior, LPA, MVPA) and phenotypic age, a weighted linear regression model was employed, with adjustment for covariates in three models: Crude model adjusted for no covariate; Model 1 was adjusted for age, sex, and race, while Model 2 (fully adjusted model) incorporated additional covariates including marital status, education, poverty status, body mass index, smokers, alcohol drinkers, hypertension, DM, and CVD. Furthermore, given that the phenotypic age index was calculated based on chronological age, we conducted a sensitivity analysis (Model 3) using covariates in Model 2, with the exception of age. In the context of our study, the regression coefficient beta (β) represented the strength and direction of the relationship between the independent variable (e.g., physical activity levels) and the dependent variable (e.g., phenotypic age). A larger absolute value of the regression coefficient beta indicated a stronger relationship between the variables, while the sign of the beta coefficient indicated the direction of the relationship (positive or negative). Subgroup and stratified analyses were then conducted to further explore these relationships under different influencing factors. Tests for linear trends across quartiles of exposures were computed by including a variable with the value for each quartile of sedentary behavior, LPA, and MVPA in the weighted linear regression models.

In addition, Isotemporal Substitution effects of different physical activity patterns on phenotypic age were further examined. The Isotemporal Substitution Model was increasingly used in physical activity and health research [37]. As a statistical approach, it was used to estimate the effect of changing the amount or type of physical activity on a health outcome, while keeping the total amount of physical activity constant. The model assumed that an individual's total amount of physical activity remained unchanged and that any change in physical activity was due to a substitution of one type of physical activity for another [27]. In a cross-sectional study design, the model was used to evaluate the effect of different physical activities on health outcomes by estimating the effect of replacing time spent in one physical activity with time spent in another physical activity.

**Fig. 2:**
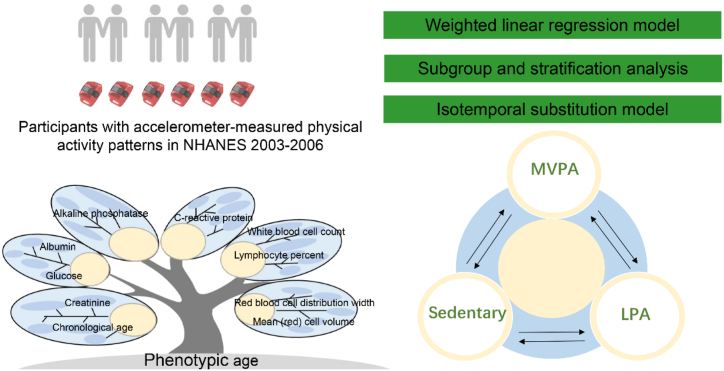
Brief introduction of the study design and analytic methods.

According to previous settings, all activity modes (i.e. sedentary behavior, light physical activity (LPA), and moderate-to-vigorous physical activity (MVPA)) were divided by a constant of 30, so as to render them equivalent to 30-min time units in accordance with physical activity guidelines [38]. Thus, each increment of one unit represented an exchange of 30 min of these behaviors per day. A variable representing the total accelerometer wear time was constructed by summing sedentary behavior, LPA and MVPA, in order to facilitate a time-substitution model. The formula for the time-substitution model was: Model = (β1) Total Time + (β2) LPA + (β3) MVPA + (β4) covariates. The model did not incorporate the variable to be substituted [39], i.e. in the above formula, the effect of an increase of 30 min of different activities on the outcome variable, with β1 – β4 representing the coefficients of different activities on health outcomes, in the absence of sedentary behavior time. Fig. 2 shows the study design, including study participants, analytic methods, composition of calculating phenotypic age, and a schematic diagram of the Isotemporal Substitution Model.

## 3 Results

**Fig. 3:**
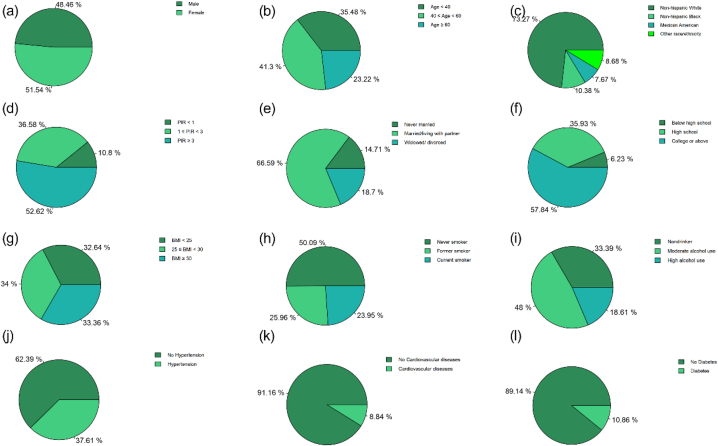
Demographic distribution of study participants. Notes: Subgraphs a-l represent the distribution of sex, age, race, poverty status, marital status, education, body mass index, smokers, alcohol drinkers, hypertension, cardiovascular disease, and diabetes mellitus in the study sample in sequence.

A total of 6439 participants were included in the final analysis, representing 49,964,300 non-institutionalized residents of the United States (Table 1). Of these, 48.46% were male and 51.54% were female, with an average age of 47.13 years. The phenotypic age was calculated based on biological markers, and the average phenotypic age was 43.05 years. The average sedentary behavior time was 476.02 min/day, with an average of 255.92 min/day spent in light physical activity (LPA) and 23.57 min/day spent in moderate-to-vigorous physical activity (MVPA). More details about the demographic information about study participants are shown in Fig. 3 (subgraphs a-l).

**Table 1:** Basic characteristics of participants in NHANES.

Variable	%
Chronological age	
<40	35.48
[40, 60)	41.30
≥60	23.22
Sex	
Male	48.46
Female	51.54
Race/ethnicity	
Non-hispanic White	73.27
Non-hispanic Black	10.38
Mexican American	7.67
Other race/ethnicity	8.68
Marital status	
Never married	14.71
Married/living with partner	66.60
Widowed/divorced	18.70
Poverty income ratio	
<1	10.80
[1,3)	36.58
≥3	52.62
Education	
Below high school	6.23
High school	35.93
College or above	57.84
BMI (kg/m^2^)	
<25	32.64
[25, 30)	34.00
≥30	33.36
Smokers	
Never smoker	50.09
Former smoker	25.96
Current smoker	23.95
Alcohol drinkers	
Nondrinker	33.39
Moderate alcohol use	48.00
High alcohol use	18.61
Hypertension	
No	62.39
Yes	37.61
Cardiovascular diseases	
No	91.16
Yes	8.84
Diabetes mellitus	
No	89.14
Yes	10.86
Sedentary behavior (as category)	
Q1 (67.5–395.7 min/day)	24.72
Q2 (395.7–478.1 min/day)	26.17
Q3 (478.1–558.2 min/day)	25.45
Q4 (558.2–1088.3 min/day)	23.66
LPA (as category)	
Q1 (13–207.4 min/day)	23.81
Q2 (207.4–254.8 min/day)	25.30
Q3 (254.8–299.9 min/day)	26.00
Q4 (299.9–608 min/day)	24.89
MVPA (as category)	
Q1 (0–4.6 min/day)	19.09
Q2 (4.6–14 min/day)	24.61
Q3 (14–30.3 min/day)	27.72
Q4 (30.3–313 min/day)	28.58
**Variable**	**Mean ± SE**
Sedentary behavior (minutes/day)	476.02 ± 0.62
LPA (minutes/day)	255.92 ± 1.22
MVPA (minutes/day)	23.57 ± 1.73
Phenotypic age (year)	43.05 ± 0.46
Chronological age (year)	47.13 ± 0.43
Red blood cell distribution width (%)	12.67 ± 0.02
Mean (red) cell volume (fL)	90.42 ± 0.13
Lymphocyte percent (%)	30.00 ± 0.13
White blood cell count (1000 cells/uL)	7.29 ± 0.04
Alkaline phosphatase (U/L)	68.32 ± 0.51
C-reactive protein (mg/dL)	0.42 ± 0.01
Albumin (g/L)	42.64 ± 0.09
Glucose (mmol/L)	5.70 ± 0.04
Creatinine (umol/L)	80.70 ± 0.41

Notes: For categorical variables: survey-weighted percentage (%). For continuous variables: survey-weighted Mean ± SE. NHANES, National Health and Nutrition Examination Survey; BMI, body mass index; LPA, light-intensity physical activity; MVPA, moderate-to-vigorous physical activity.

Considering the complex multistage sampling design, the weighted linear regression model was applied to explore the relationship between sedentary behavior, LPA, MVPA and phenotypic age (Table 2). Using the continuous measures, it was demonstrated that sedentary behavior was significantly positively associated with phenotypic age (Crude Model, β: 0.043, p < 0.001; Model 1, β: 0.010, p < 0.001; Model 2, β: 0.009, p < 0.001), while LPA (Crude Model, β: −0.036, p < 0.001; Model 1, β: −0.012, p < 0.001; Model 2, β: −0.010, p < 0.001) and MVPA (Crude Model, β: −0.295, p < 0.001; Model 1, β: −0.097, p < 0.001; Model 2, β: −0.062, p < 0.001) was significantly negatively associated with phenotypic age.

**Table 2:** Associations between accelerometer-measured physical activity patterns and phenotypic age.

	Crude model^a^		Model 1^b^		Model 2^c^	
	β (95% CI)	*p-value*	β (95% CI)	*p-value*	β (95% CI)	*p-value*
Sedentary behavior (minutes/day)	0.043(0.037,0.048)	<0.001	0.010(0.007, 0.013)	<0.001	0.009(0.007, 0.011)	<0.001
Sedentary behavior (as category)						
Q1 (67.5–395.7 min/day)	Reference		Reference		Reference	
Q2 (395.7–478.1 min/day)	3.998(2.254, 5.743)	<0.001	0.315(-0.479, 1.109)	0.420	0.661(-0.052, 1.373)	0.066
Q3 (478.1–558.2 min/day)	8.989(7.369,10.610)	<0.001	1.442(0.676, 2.208)	<0.001	1.532(0.767, 2.297)	0.001
Q4 (558.2–1088.3 min/day)	13.082(11.021,15.143)	<0.001	3.117(2.172, 4.061)	<0.001	2.993(2.204, 3.782)	<0.001
LPA (minutes/day)	−0.036(-0.049,-0.024)	<0.001	−0.012(-0.017,-0.008)	<0.001	−0.010(-0.013,-0.006)	<0.001
LPA (as category)						
Q1 (13–207.4 min/day)	Reference		Reference		Reference	
Q2 (207.4–254.8 min/day)	−4.477(-6.517,-2.437)	<0.001	−1.845(-2.594,-1.095)	<0.001	−1.269(-1.877,-0.660)	<0.001
Q3 (254.8–299.9 min/day)	−6.115(-8.264,-3.966)	<0.001	−1.809(-2.730,-0.889)	<0.001	−1.232(-2.124,-0.341)	0.012
Q4 (299.9–608 min/day)	−6.500(-8.876,-4.125)	<0.001	−1.987(-2.815,-1.159)	<0.001	−1.476(-2.146,-0.805)	<0.001
MVPA (minutes/day)	−0.295(-0.321,-0.270)	<0.001	−0.097(-0.111,-0.082)	<0.001	−0.062(-0.075,-0.048)	<0.001
MVPA (as category)						
Q1 (0–4.6 min/day)	Reference		Reference		Reference	
Q2 (4.6–14 min/day)	−16.430(-18.058,-14.801)	<0.001	−5.187(−6.031,-4.343)	<0.001	−3.723(-4.552,-2.893)	<0.001
Q3 (14–30.3 min/day)	−22.318(-23.991,-20.646)	<0.001	−7.384(−8.544,-6.223)	<0.001	−5.003(-6.004,-4.003)	<0.001
Q4 (30.3–313 min/day)	−26.743(-28.825,-24.661)	<0.001	−9.247(-10.421,-8.074)	<0.001	−6.216(-7.292,-5.139)	<0.001

Notes: ^a^Crude model, no covariate was adjusted. ^b^Model 1, age, sex, race were adjusted. ^c^Model 2, age, sex, race, marital status, education, poverty status, body mass index, smokers, alcohol drinkers, hypertension, diabetes mellitus, and cardiovascular diseases were adjusted. CI, confidence interval; LPA, light-intensity physical activity; MVPA, moderate-to-vigorous physical activity.

As for the categorized (quantile) measures, which were consistent with those in Table 1, our results identified a similar trend, either using the crude or the adjusted model (Table 2). Compared with the first quantile, participants with the fourth quantile of sedentary behavior gained 2.99 years increase in the phenotypic age [β: 2.993, p < 0.001] in the fully adjusted model. However, taking the first quantile as a reference, participants with the fourth quantile of LPA and MVPA decreased 1.48 years and 6.22 years’ phenotypic age (β: −1.476, p < 0.001; β: −6.216, p < 0.001), respectively. Sensitivity analysis (Table S1) was conducted using Model 3, which was adjusted for covariates in Model 2 except for age, and the results were consistent with findings in Crude model, Model 1 and Model 2.

**Fig. 4:**
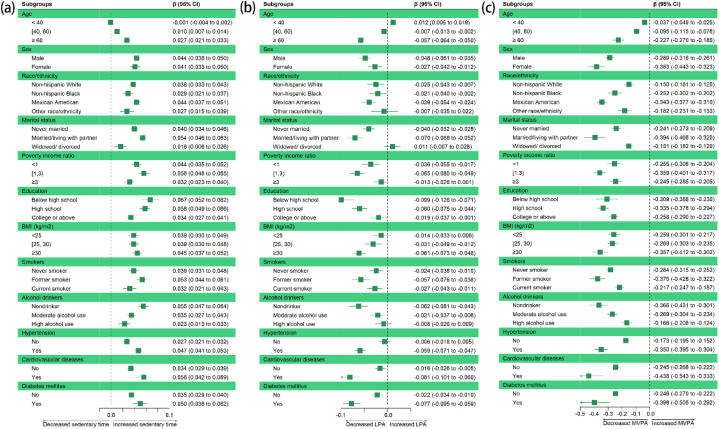
Forest plot of subgroup analysis between sedentary time (a), light-intensity physical activity (LPA) (b), moderate-to-vigorous physical activity (MVPA) (c) and phenotypic age. Crude Model was used for the subgroup analysis.

Additionally, the subgroup analysis showed that the associations between sedentary time (Fig. 4a), LPA (Fig. 4b), MVPA (Fig. 4c) and phenotypic age were consistent at different levels of influencing factors. When categorized by chronological age, it was observed that the elderly group (≥60 years) was more vulnerable to alterations in physical activity patterns, with a larger effect size (for sedentary behavior, β: 0.027; for LPA, β: −0.057; for MVPA, β: −0.227). An increase of 1 min per day in sedentary behavior in this group was associated with an increase of 0.03 years in phenotypic age, while an increase of 1 min per day in LPA and MVPA was associated with decrease of 0.06 and 0.23 years in phenotypic age, respectively, indicating beneficial effects of engaging LPA and MVPA.

It was also necessary to consider chronic diseases as important influencing factors. Our results showed that in groups with hypertension, participants had increased 0.05 years of phenotypic age with a 1-min per day increment in sedentary behavior. Moreover, those with hypertension could benefit from LPA and MVPA with decreased 0.06 and 0.35 years of phenotypic age with 1-min per day increment in each type of physical activity. For participants with cardiovascular diseases, it was demonstrated that each additional minute of sedentary behavior per day was associated with an increase of 0.06 years of their phenotypic age. In contrast, for individuals with cardiovascular diseases, a 1-min increment in LPA and MVPA was associated with a decrease in phenotypic age of 0.08 and 0.44 years. For diabetes mellitus patients, a 1-min per day increment in sedentary behavior was associated with increased 0.05 years of phenotypic age, while 1-min per day increment in LPA and MVPA was associated with decreased 0.08 and 0.40 years of phenotypic age, respectively. Moreover, stratified analysis using the quantile of sedentary time (Table S2), LPA (Table S3), MVPA (Table S4) verified that these associations were consistent across the different subgroups.

Furthermore, the results of the Isotemporal Substitution Model (Table 3) reported the influence of replacement of sedentary behavior, LPA, and MVPA on phenotypic age. It was shown that replacing 30 min of sedentary time with either LPA or MVPA was associated with decreased phenotypic age, with β values of −0.355 and −1.892, respectively (p < 0.001). Conversely, substituting 30 min of LPA with the same amount of sedentary time was found to be associated with increased phenotypic age (β: 0.296, p < 0.001), while replacing 30 min of LPA with the same amount of MVPA was associated with decreased phenotypic age (β: −1.285, p < 0.001). Additionally, 30 min sedentary behavior or LPA isochronous replacement of MVPA was associated with increased phenotypic age (β: 0.741, p < 0.001; β: 0.616, p < 0.001).

**Table 3:** Effects of 30 min/day replacement of physical activity patterns on phenotypic age using the Isotemporal Substitution Model.

	Sedentary behavior		LPA		MVPA	
	β (95% CI)	*p-value*	β (95% CI)	*p-value*	β (95% CI)	*p-value*
Sedentary behavior (30 * minutes/day)	Dropped		−0.355(-0.483,-0.227)	<0.001	−1.892(-2.300,-1.485)	<0.001
LPA (30 * minutes/day)	0.296(0.205, 0.388)	<0.001	Dropped		−1.285(-1.671,-0.900)	<0.001
MVPA (30 * minutes/day)	0.741(0.573, 0.908)	<0.001	0.616(0.390, 0.843)	<0.001	Dropped	

Notes: Fully adjusted model (Model 2) was used. Age, sex, race, marital status, education, poverty status, body mass index, smokers, alcohol drinkers, hypertension, diabetes mellitus, and cardiovascular diseases were adjusted. CI, confidence interval; LPA, light-intensity physical activity; MVPA, moderate-to-vigorous physical activity.

## 4 Discussions

The results of this study on a nationally representative sample of adult U.S. citizens revealed two main findings. First, we found that sedentary behavior exhibited a positive association with phenotypic age, while physical activity, including both light physical activity (LPA) and moderate-to-vigorous physical activity (MVPA), showed a negative association with phenotypic age. Second, by using the Isotemporal Substitution Model, it was important to note that negative associations between sedentary behavior and phenotypic age can be mitigated by engaging in regular physical activity (especially MVPA) and breaking up prolonged periods of sitting. A subgroup and stratified analysis confirmed the main analysis results, reinforcing our confidence in the aforementioned results.

This study demonstrated that sedentary lifestyle was a risk factor associated with the aging process, which may suggest that more time spent on prolonged sitting was related to accelerated aging. This was consistent with one of the latest findings detecting the relationship between habitual sedentary time and epigenetic age [40]. However, another cross-sectional study in the elder group found that there was no significant association between biological age (indexed by epigenetic age acceleration) and sedentary time [41]. This might be attributed to the small study subjects (less than 300 samples) and the differences in the selection of biological indicators. There have been a number of underlying pathways suggested by scholars that sedentary behavior might affect aging. Basically, sedentary behavior was linked to changes in gene expression, including upregulation of genes associated with aging and downregulation of genes involved in tissue repair [42]. One study showed that 60 days of bed rest caused ribonucleic acid (RNA) repair and damage transcripts in muscle tissue to be upregulated in young women [43]. Moreover, there was also evidence that sedentary behavior was associated with alterations in epigenetic pathways, such as downregulation of MicroRNAs associated with cardiac function and skeletal muscle atrophy [44].

In line with previous studies [45,46], our study suggested that performing regular physical activity was negatively associated with aging process and in particular the biological age of an individual. Moreover, subgroup analysis of participants with different chronic diseases showed a positive impact of physical activity on slowing aging. These findings provided novel insights that supported the progress of international physical activity guidelines for the high-risk population. One study confirmed our results among adults with diabetes and found that both LPA and MVPA could reduce biomarkers including C-reactive protein and triglycerides [47]. Another UK biobank study also identified that accelerometer-assessed physical activity was negatively associated with mortality and incidence of cardiovascular disease among adults with hypertension [48]. In addition, replacement of 30 min’ sedentary behavior with MVPA was found to be correlated with substantial decreases in both systolic and diastolic blood pressure as well as diminishing body mass index [49].

Furthermore, we complemented the existing literature and sought to establish a dose-response relation between physical activity and phenotypic age. From our results, MVPA had expanded benefits than LPA on the association between phenotypic age and using 30 min MVPA to replace sedentary time was associated with 1.89 years’ decrease in phenotypic age. Moreover, this trend was also identified in groups with chronic diseases. It was suggested that in remedying overweight, high intensity exercise led to better changes of body composition and took less time than light or moderate exercise to achieve comparable results [50,51]. Scholars also found that high intensity exercise favored better reductions in post-prandial hyperglycemia [52] and was associated with improved hypertension control [53] when compared to moderate exercise.

The biological mechanisms underlying physical activity that mitigated phenotypic aging were complex and not fully understood. One of the most widely accepted theories was that physical activity reduces inflammation [54,55]. Inflammation is a natural process that occurs in response to injury or infection, but can also contribute to the aging process [19]. Regular physical exercise has been shown to change the leukocytes’ phenotype and increase the production of interleukin-6 (IL-6) and interleukin-10 (IL-10) from skeletal muscle [56,57], which in turn can reduce the rate of biological aging. In recent years, there was also mounting evidence that highlighted the increasing accuracy of epigenetic clocks, on the basis of DNA methylation (DNAm) measures in predicting biological age and their relationship with physical activity [58]. In detail, increasing physical activity might slow down aging by increasing antioxidant capacity and reducing reactive oxygen species, both of which result in a higher repair capacity for DNA [59]. When it comes to the substitution effect of physical activity on sedentary behavior, a recent animal study obtained 'runner plasma' from the exercising mice and shifted it to mice who were sedentary and found that neuro plasticity and brain inflammation was then significantly improved in the sedentary mice [60]. This interesting finding further supported our population-based results from a biological perspective.

## 5 Strengths and limitations

Our findings had significant implications for aging management and public health issues, which emphasized the importance of maintaining certain volume of physical exercise and reducing sedentary time. This study was a strong result of utilizing the nationally representative NHANES sample, which highlighted the importance of considering the influence of lifestyle behavior such as physical activity patterns on phenotypic age. Additionally, objectively measured physical activity based on accelerometers and application of the Isotemporal Substitution Model increased the robustness of study findings and enriched the research information. Besides, we used weighted analysis in consideration of the complex multistage sampling design and conducted subgroup analyses to produce more reliable results. This study also took chronic diseases into account when considering the physical activity's influence on aging.

Several shortcomings of the study should be acknowledged. Primarily, our research was founded upon an analysis of a cross-sectional NHANES dataset, thus we cannot deduce the causative inference. Despite the utilization of extensive samples from a nationally representative survey, it is essential to acknowledge the possibility of unaccounted variables (such as dietary habits, stress, depression & psychological disorders, obesity and other conditions) that could have influenced the study findings. Moreover, the phenotypic age index was calculated based on chronological age and several biomarkers. Hence, confounding factors adjusted in the regression model had a risk of over adjustment. However, we conducted a sensitivity analysis to mitigate this potential shortcoming. The chosen NHANES dataset timeframe is not the most recent (2003/2004 and 2005/2006), thus further study could use the latest data to verify our findings. Furthermore, the confinement of our study to the United States population limited the extrapolation of our results to other demographics, although the complex multistage sampling partially compensated for the deficiency.

## 6 Conclusion

Using a nationwide population, this study firstly identified the relationship between accelerometer-measured physical activity patterns and an emerging phenotypic age index. The findings of this study found that prolonged sedentary time was associated with elevated phenotypic age. An elevated phenotypic age may indicate accelerated aging, and this could be associated with an increased risk of age-related diseases and mortality. As such, interventions such as physical activity targeting sedentary behavior may be beneficial for reducing the negative impacts of sedentary behavior on the biological aging process. More importantly, performing 30 min of MVPA in a day to replace sedentary time was associated with 1.9 years decrease of phenotypic age. This information can be useful for predicting future health outcomes, determining risk for age-related diseases, and guiding the development of personalized health and wellness plans. Randomized controlled trials, prospective cohort research, and Mendelian randomization studies are encouraged to confirm and expand our results.

## Author contribution statement

Yanwei You and Yuquan Chen: Conceived and designed the experiments; Performed the experiments; Analyzed and interpreted the data; Contributed reagents, materials, analysis tools or data; Wrote the paper.

Xiaoxin Wang: Conceived and designed the experiments; Performed the experiments; Analyzed and interpreted the data; Wrote the paper.

Mengxian Wei and Qi Zhang: Contributed reagents, materials, analysis tools or data; Wrote the paper.

Qiang Cao: Conceived and designed the experiments; Contributed reagents, materials, analysis tools or data.

### 6.1 Data availability statement

Data associated with this study has been deposited at https://www.cdc.gov/nchs/nhanes/.

## Funding statement

This research received no external funding.

## 7 Institutional review board statement

Research procedures of NHANES was approved by the Institutional Review Board (IRB) of the National Center for Health Statistics (NCHS). Written informed consents were obtained from all participants.

## Declaration of competing interest

The authors declare that they have no known competing financial interests or personal relationships that could have appeared to influence the work reported in this paper.
