# Supplementary material for: Accelerometer-measured physical activity patterns are associated with phenotypic age: Isotemporal substitution effects
Source: Heliyon. 2023 Aug 23;9(9):e19158. doi: 10.1016/j.heliyon.2023.e19158 (PMC10558316; doi:10.1016/j.heliyon.2023.e19158)
Supplement: Multimedia component 1 [file mmc1.docx]

Table S1. Sensitivity analysis of associations between accelerometer-measured physical activity patterns and phenotypic age.

|  |  |  | Model 3 | |
| --- | --- | --- | --- | --- |
|  |  |  | β (95% CI) | *p-value* |
| Sedentary behavior (minutes/day) |  |  | 0.025( 0.021, 0.030) | <0.001 |
| Sedentary behavior (as category) |  |  |  |  |
| Q1 (67.5-395.7 minutes/day) |  |  | Reference |  |
| Q2 (395.7-478.1 minutes/day) |  |  | 2.63( 1.450, 3.809) | <0.001 |
| Q3 (478.1-558.2 minutes/day) |  |  | 5.215( 3.780, 6.651) | <0.001 |
| Q4 (558.2-1088.3 minutes/day) |  |  | 8.193( 6.867, 9.518) | <0.001 |
| LPA (minutes/day) |  |  | -0.018(-0.025,-0.012) | <0.001 |
| LPA (as category) |  |  |  |  |
| Q1 (13-207.4 minutes/day) |  |  | Reference |  |
| Q2 (207.4-254.8 minutes/day) |  |  | -2.198(-3.289,-1.107) | <0.001 |
| Q3 (254.8-299.9 minutes/day) |  |  | -3.023(-4.332,-1.713) | <0.001 |
| Q4 (299.9-608 minutes/day) |  |  | -3.115(-4.393,-1.837) | <0.001 |
| MVPA (minutes/day) |  |  | -0.158(-0.179,-0.138) | <0.001 |
| MVPA (as category) |  |  |  |  |
| Q1 (0-4.6 minutes/day) |  |  | Reference |  |
| Q2 (4.6-14 minutes/day) |  |  | -9.304(-10.839, -7.768) | <0.001 |
| Q3 (14-30.3 minutes/day) |  |  | -12.735(-14.170,-11.300) | <0.001 |
| Q4 (30.3-313 minutes/day) |  |  | -15.689(-17.636,-13.741) | <0.001 |

Notes: Model 3, sex, race, marital status, education, poverty status, body mass index, smokers, alcohol drinkers, hypertension, diabetes mellitus, and cardiovascular diseases were adjusted. This is a sensitivity analysis for Table 2 (covariates in Model 3 were the same as Model 2 except for chronological age). CI, confidence interval; LPA, light-intensity physical activity; MVPA, moderate-to-vigorous physical activity.

Table S2. Weighted linear regression of stratified results for associations between sedentary behavior and phenotypic age.

|  | Q1 | Q2 | *p* | Q3 | *p* | Q4 | *p* | ptrend |
| --- | --- | --- | --- | --- | --- | --- | --- | --- |
| Age |  |  |  |  |  |  |  |  |
| < 40 | Reference | -0.394(-1.564,0.777) | 0.496 | -0.34(-1.332,0.651) | 0.488 | -0.469(-1.458,0.520) | 0.339 | 0.328 |
| [40, 60) | Reference | 0.934(-0.039,1.907) | 0.059 | 2.495( 1.598,3.392) | <0.001 | 3.167( 1.926,4.408) | <0.001 | <0.001 |
| ≥ 60 | Reference | 2.046(0.329, 3.763) | 0.021 | 4.334(2.495, 6.172) | <0.001 | 8.736(6.657,10.816) | <0.001 | <0.001 |
| Sex |  |  |  |  |  |  |  |  |
| Male | Reference | 5.325( 3.689, 6.962) | <0.001 | 10.628( 8.826,12.429) | <0.001 | 13.899(11.978,15.820) | <0.001 | <0.001 |
| Female | Reference | 2.678(0.042, 5.313) | 0.047 | 7.439(5.280, 9.598) | <0.001 | 12.304(9.145,15.462) | <0.001 | <0.001 |
| Race/ethnicity |  |  |  |  |  |  |  |  |
| Non-hispanic White | Reference | 5.257( 3.301, 7.213) | <0.001 | 8.595( 5.755,11.434) | <0.001 | 13.594(11.097,16.091) | <0.001 | <0.001 |
| Non-hispanic Black | Reference | 3.44(1.267, 5.613) | 0.003 | 5.734(3.516, 7.952) | <0.001 | 9.606(6.524,12.687) | <0.001 | <0.001 |
| Mexican American | Reference | 3.291( 0.722, 5.860) | 0.014 | 9.042( 6.959,11.126) | <0.001 | 12.905(10.346,15.464) | <0.001 | <0.001 |
| Other race/ethnicity | Reference | 4.155(1.295, 7.015) | 0.006 | 4.993(0.432, 9.554) | 0.033 | 9.445(4.432,14.458) | <0.001 | <0.001 |
| Marital status |  |  |  |  |  |  |  |  |
| Never married | Reference | 3.535(2.072, 4.998) | <0.001 | 8.363(6.880, 9.845) | <0.001 | 12.024(9.917,14.130) | <0.001 | <0.001 |
| Married/living with partner | Reference | 6.201( 3.123, 9.278) | <0.001 | 12.426(10.004,14.848) | <0.001 | 18.202(14.687,21.717) | <0.001 | <0.001 |
| Widowed/ divorced | Reference | 0.544(-2.080,3.168) | 0.674 | 3.331( 0.386,6.275) | 0.028 | 4.663( 1.239,8.086) | 0.009 | 0.003 |
| Poverty income ratio |  |  |  |  |  |  |  |  |
| < 1 | Reference | 3.57( 0.801, 6.340) | 0.013 | 12.127( 8.973,15.281) | <0.001 | 16.072(12.385,19.758) | <0.001 | <0.001 |
| [1,3) | Reference | 5.246( 3.117, 7.374) | <0.001 | 12.711(10.159,15.263) | <0.001 | 18.891(15.636,22.146) | <0.001 | <0.001 |
| ≥ 3 | Reference | 2.7(0.459, 4.941) | 0.020 | 5.63(3.549, 7.710) | <0.001 | 8.799(6.041,11.557) | <0.001 | <0.001 |
| Education |  |  |  |  |  |  |  |  |
| Below high school | Reference | 10.626( 6.464,14.787) | <0.001 | 20.702(15.902,25.501) | <0.001 | 26.699(21.646,31.751) | <0.001 | <0.001 |
| High school | Reference | 5.547( 3.549, 7.545) | <0.001 | 13.42(10.666,16.175) | <0.001 | 19.411(16.288,22.535) | <0.001 | <0.001 |
| College or above | Reference | 3.031(0.769, 5.292) | 0.011 | 6.637(4.602, 8.671) | <0.001 | 9.774(7.286,12.262) | <0.001 | <0.001 |
| BMI (kg/m^2^) |  |  |  |  |  |  |  |  |
| < 25 | Reference | 2.788(-0.693, 6.269) | 0.112 | 7.908( 5.197,10.619) | <0.001 | 10.514( 6.970,14.058) | <0.001 | <0.001 |
| [25, 30) | Reference | 4.681( 2.976, 6.385) | <0.001 | 9.381( 6.971,11.792) | <0.001 | 12.751(10.195,15.308) | <0.001 | <0.001 |
| ≥ 30 | Reference | 4.502( 2.016, 6.988) | <0.001 | 9.366( 7.080,11.652) | <0.001 | 14.477(11.776,17.179) | <0.001 | <0.001 |
| Smokers |  |  |  |  |  |  |  |  |
| Never smoker | Reference | 2.987(1.053, 4.921) | 0.004 | 8.069(5.786,10.353) | <0.001 | 11.954(9.187,14.720) | <0.001 | <0.001 |
| Former smoker | Reference | 4.665( 1.689, 7.642) | 0.003 | 9.225( 6.485,11.966) | <0.001 | 15.634(12.621,18.647) | <0.001 | <0.001 |
| Current smoker | Reference | 3.868(1.075, 6.660) | 0.008 | 8.852(5.590,12.113) | <0.001 | 10.58(7.076,14.084) | <0.001 | <0.001 |
| Alcohol drinkers |  |  |  |  |  |  |  |  |
| Nondrinker | Reference | 5.201( 2.400, 8.002) | <0.001 | 10.749( 7.930,13.568) | <0.001 | 17.741(14.345,21.137) | <0.001 | <0.001 |
| Moderate alcohol use | Reference | 3.481(1.146, 5.815) | 0.005 | 7.836(5.537,10.135) | <0.001 | 10.328(7.684,12.971) | <0.001 | <0.001 |
| High alcohol use | Reference | 1.883(-0.397, 4.163) | 0.102 | 4.912( 1.698, 8.127) | 0.004 | 7.285( 4.056,10.514) | <0.001 | <0.001 |
| Hypertension |  |  |  |  |  |  |  |  |
| No | Reference | 2.494(0.920,4.067) | 0.003 | 5.622(3.757,7.488) | <0.001 | 7.882(6.052,9.713) | <0.001 | <0.001 |
| Yes | Reference | 4.41( 2.313, 6.507) | <0.001 | 8.696( 6.571,10.821) | <0.001 | 15.014(12.727,17.301) | <0.001 | <0.001 |
| Cardiovascular diseases |  |  |  |  |  |  |  |  |
| No | Reference | 3.455(1.700, 5.210) | <0.001 | 7.466(5.955, 8.977) | <0.001 | 10.263(8.396,12.130) | <0.001 | <0.001 |
| Yes | Reference | 7.606( 2.765,12.447) | 0.003 | 11.327( 6.690,15.965) | <0.001 | 20.076(15.521,24.632) | <0.001 | <0.001 |
| Diabetes mellitus |  |  |  |  |  |  |  |  |
| No | Reference | 3.717(2.060, 5.373) | <0.001 | 7.678(6.055, 9.301) | <0.001 | 10.462(8.545,12.379) | <0.001 | <0.001 |
| Yes | Reference | 4.368(-1.653,10.388) | 0.148 | 9.272( 5.351,13.192) | <0.001 | 16.347(11.597,21.097) | <0.001 | <0.001 |

Notes: Q1 of sedentary behavior was used as reference. Q1 (67.5-395.7 minutes/day), Q2 (395.7-478.1 minutes/day), Q3 (478.1-558.2 minutes/day), Q4 (558.2-1088.3 minutes/day); Abbreviations: BMI, body mass index; ptrend, p for trend.

Table S3. Weighted linear regression of stratified results for associations between light physical activity (LPA) and phenotypic age.

|  | Q1 | Q2 | *p* | Q3 | *p* | Q4 | *p* | ptrend |
| --- | --- | --- | --- | --- | --- | --- | --- | --- |
| Age |  |  |  |  |  |  |  |  |
| < 40 | Reference | 0.222(-0.960,1.405) | 0.702 | 1.11(-0.079,2.299) | 0.066 | 2.015( 0.612,3.417) | 0.007 | 0.003 |
| [40, 60) | Reference | -0.319(-1.808,1.169) | 0.663 | -0.248(-1.858,1.363) | 0.755 | -1.016(-2.177,0.145) | 0.084 | 0.097 |
| ≥ 60 | Reference | -5.857( -7.126,-4.589) | <0.001 | -7.667( -9.047,-6.286) | <0.001 | -9.296(-10.614,-7.978) | <0.001 | <0.001 |
| Sex |  |  |  |  |  |  |  |  |
| Male | Reference | -4.96( -7.081,-2.839) | <0.001 | -7.140( -9.518,-4.763) | <0.001 | -8.301(-10.647,-5.954) | <0.001 | <0.001 |
| Female | Reference | -3.909(-6.815,-1.002) | 0.010 | -5.063(-7.901,-2.225) | 0.001 | -5.049(-8.058,-2.040) | 0.002 | 0.001 |
| Race/ethnicity |  |  |  |  |  |  |  |  |
| Non-hispanic White | Reference | -5.763( -8.890,-2.635) | <0.001 | -5.906( -8.860,-2.953) | <0.001 | -6.636(-10.313,-2.958) | 0.001 | 0.009 |
| Non-hispanic Black | Reference | -3.975(-7.346,-0.603) | 0.023 | -1.348(-4.386, 1.691) | 0.368 | -3.619(-7.651, 0.413) | 0.076 | 0.181 |
| Mexican American | Reference | -4.661(-6.862,-2.461) | <0.001 | -6.795(-9.251,-4.338) | <0.001 | -6.787(-9.495,-4.079) | <0.001 | <0.001 |
| Other race/ethnicity | Reference | -0.067(-6.657,6.523) | 0.984 | -1.295(-6.662,4.072) | 0.625 | -0.054(-5.657,5.549) | 0.984 | 0.888 |
| Marital status |  |  |  |  |  |  |  |  |
| Never married | Reference | -3.781(-5.962,-1.601) | 0.001 | -6.358(-8.641,-4.075) | <0.001 | -7.016(-9.465,-4.567) | <0.001 | <0.001 |
| Married/living with partner | Reference | -10.541(-13.883, -7.199) | <0.001 | -11.541(-15.132, -7.950) | <0.001 | -13.728(-17.344,-10.113) | <0.001 | <0.001 |
| Widowed/ divorced | Reference | -0.127(-3.569,3.315) | 0.940 | 1.296(-1.653,4.244) | 0.375 | 2.030(-1.817,5.877) | 0.289 | 0.244 |
| Poverty income ratio |  |  |  |  |  |  |  |  |
| < 1 | Reference | -6.111(-10.732,-1.490) | 0.011 | -8.573(-12.774,-4.372) | <0.001 | -6.934(-10.919,-2.949) | 0.001 | <0.001 |
| [1,3) | Reference | -8.102(-11.345,-4.860) | <0.001 | -11.426(-15.053,-7.798) | <0.001 | -12.035(-15.390,-8.680) | <0.001 | <0.001 |
| ≥ 3 | Reference | -1.948(-4.145, 0.249) | 0.080 | -2.392(-4.700,-0.084) | 0.043 | -2.744(-5.294,-0.194) | 0.036 | 0.041 |
| Education |  |  |  |  |  |  |  |  |
| Below high school | Reference | -13.388(-21.068, -5.708) | 0.001 | -15.42(-22.630, -8.211) | <0.001 | -20.002(-25.830,-14.174) | <0.001 | <0.001 |
| High school | Reference | -9.523(-13.242,-5.804) | <0.001 | -11.93(-15.589,-8.272) | <0.001 | -12.709(-16.253,-9.165) | <0.001 | <0.001 |
| College or above | Reference | -1.597(-3.692, 0.497) | 0.129 | -3.254(-5.786,-0.722) | 0.014 | -3.029(-6.144, 0.086) | 0.056 | 0.037 |
| BMI (kg/m^2^) |  |  |  |  |  |  |  |  |
| < 25 | Reference | -0.809(-4.790,3.172) | 0.680 | -2.316(-6.114,1.483) | 0.222 | -1.839(-5.692,2.015) | 0.336 | 0.220 |
| [25, 30) | Reference | -4.423(-7.063,-1.783) | 0.002 | -5.365(-9.092,-1.639) | 0.006 | -5.898(-8.984,-2.811) | <0.001 | 0.001 |
| ≥ 30 | Reference | -7.498( -9.722,-5.274) | <0.001 | -9.588(-11.706,-7.470) | <0.001 | -10.815(-13.084,-8.545) | <0.001 | <0.001 |
| Smokers |  |  |  |  |  |  |  |  |
| Never smoker | Reference | -2.875(-5.550,-0.200) | 0.036 | -3.912(-6.516,-1.308) | 0.005 | -3.986(-6.679,-1.294) | 0.005 | 0.003 |
| Former smoker | Reference | -6.254( -9.271,-3.237) | <0.001 | -9.233(-12.962,-5.505) | <0.001 | -9.587(-12.775,-6.399) | <0.001 | <0.001 |
| Current smoker | Reference | -3.644(-6.843,-0.444) | 0.027 | -4.388(-7.092,-1.684) | 0.003 | -5.432(-8.325,-2.539) | <0.001 | <0.001 |
| Alcohol drinkers |  |  |  |  |  |  |  |  |
| Nondrinker | Reference | -6.981( -9.977,-3.985) | <0.001 | -10.011(-13.252,-6.771) | <0.001 | -10.739(-14.419,-7.060) | <0.001 | <0.001 |
| Moderate alcohol use | Reference | -2.063(-4.494, 0.367) | 0.093 | -3.386(-5.941,-0.831) | 0.011 | -3.650(-6.407,-0.892) | 0.011 | 0.008 |
| High alcohol use | Reference | -3.422(-7.263,0.420) | 0.079 | -2.966(-6.608,0.676) | 0.106 | -2.615(-6.076,0.845) | 0.133 | 0.240 |
| Hypertension |  |  |  |  |  |  |  |  |
| No | Reference | -1.635(-4.069, 0.799) | 0.179 | -1.97(-3.922,-0.019) | 0.048 | -1.157(-3.425, 1.111) | 0.305 | 0.305 |
| Yes | Reference | -6.746( -8.695,-4.797) | <0.001 | -8.687(-11.218,-6.157) | <0.001 | -10.674(-12.670,-8.678) | <0.001 | <0.001 |
| Cardiovascular diseases |  |  |  |  |  |  |  |  |
| No | Reference | -1.692(-3.656, 0.271) | 0.088 | -2.846(-4.689,-1.003) | 0.004 | -2.896(-4.984,-0.807) | 0.008 | 0.005 |
| Yes | Reference | -9.886(-12.352,-7.420) | <0.001 | -12.848(-18.333,-7.364) | <0.001 | -12.383(-17.006,-7.761) | <0.001 | <0.001 |
| Diabetes mellitus |  |  |  |  |  |  |  |  |
| No | Reference | -2.310(-4.273,-0.347) | 0.023 | -3.752(-5.810,-1.693) | <0.001 | -3.651(-5.963,-1.340) | 0.003 | 0.002 |
| Yes | Reference | -9.465(-12.974, -5.957) | <0.001 | -9.298(-12.902, -5.694) | <0.001 | -15.042(-19.235,-10.849) | <0.001 | <0.001 |

Notes: Q1 of light physical activity (LPA) was used as reference. Q1 (13-207.4 minutes/day), Q2 (207.4-254.8 minutes/day), Q3 (254.8-299.9 minutes/day), Q4 (299.9-608 minutes/day); Abbreviations: BMI, body mass index; ptrend, p for trend.

Table S4. Weighted linear regression of stratified results for associations between moderate-to-vigorous physical activity (MVPA) and phenotypic age.

|  | Q1 | Q2 | *p* | Q3 | *p* | Q4 | *p* | ptrend |
| --- | --- | --- | --- | --- | --- | --- | --- | --- |
| Age |  |  |  |  |  |  |  |  |
| < 40 | Reference | 0.060(-1.556, 1.676) | 0.940 | -1.238(-2.886, 0.409) | 0.135 | -2.297(-3.947,-0.648) | 0.008 | <0.001 |
| [40, 60) | Reference | -2.899(-4.377,-1.422) | <0.001 | -5.230(-7.222,-3.238) | <0.001 | -7.355(-9.393,-5.317) | <0.001 | <0.001 |
| ≥ 60 | Reference | -7.761( -9.049,-6.473) | <0.001 | -10.161(-11.441,-8.881) | <0.001 | -11.168(-12.904,-9.431) | <0.001 | <0.001 |
| Sex |  |  |  |  |  |  |  |  |
| Male | Reference | -16.773(-19.337,-14.208) | <0.001 | -25.036(-27.633,-22.439) | <0.001 | -30.902(-33.867,-27.938) | <0.001 | <0.001 |
| Female | Reference | -17.163(-19.084,-15.242) | <0.001 | -21.74(-23.605,-19.876) | <0.001 | -25.404(-27.911,-22.897) | <0.001 | <0.001 |
| Race/ethnicity |  |  |  |  |  |  |  |  |
| Non-hispanic White | Reference | -20.219(-25.888,-14.550) | <0.001 | -22.777(-27.588,-17.966) | <0.001 | -26.273(-31.826,-20.720) | <0.001 | <0.001 |
| Non-hispanic Black | Reference | -16.219(-18.434,-14.003) | <0.001 | -21.472(-24.578,-18.367) | <0.001 | -26.184(-30.122,-22.246) | <0.001 | <0.001 |
| Mexican American | Reference | -15.785(-17.455,-14.115) | <0.001 | -22.267(-24.021,-20.513) | <0.001 | -26.966(-29.083,-24.850) | <0.001 | <0.001 |
| Other race/ethnicity | Reference | -14.381(-22.653, -6.109) | 0.001 | -15.944(-24.401, -7.487) | <0.001 | -19.307(-27.617,-10.996) | <0.001 | <0.001 |
| Marital status |  |  |  |  |  |  |  |  |
| Never married | Reference | -15.08(-16.758,-13.401) | <0.001 | -18.741(-20.449,-17.032) | <0.001 | -22.657(-24.984,-20.331) | <0.001 | <0.001 |
| Married/living with partner | Reference | -16.048(-18.415,-13.682) | <0.001 | -23.073(-25.381,-20.765) | <0.001 | -26.672(-29.660,-23.684) | <0.001 | <0.001 |
| Widowed/ divorced | Reference | -8.404(-15.162, -1.645) | 0.017 | -15.476(-22.543, -8.409) | <0.001 | -17.995(-24.292,-11.698) | <0.001 | <0.001 |
| Poverty income ratio |  |  |  |  |  |  |  |  |
| < 1 | Reference | -18.508(-22.338,-14.678) | <0.001 | -28.083(-31.499,-24.668) | <0.001 | -27.907(-31.653,-24.162) | <0.001 | <0.001 |
| [1,3) | Reference | -18.622(-21.242,-16.003) | <0.001 | -24.709(-27.397,-22.021) | <0.001 | -32.035(-34.574,-29.497) | <0.001 | <0.001 |
| ≥ 3 | Reference | -13.47(-15.624,-11.315) | <0.001 | -18.123(-20.618,-15.627) | <0.001 | -21.866(-24.659,-19.072) | <0.001 | <0.001 |
| Education |  |  |  |  |  |  |  |  |
| Below high school | Reference | -18.291(-21.610,-14.972) | <0.001 | -26.151(-29.732,-22.569) | <0.001 | -31.304(-35.996,-26.612) | <0.001 | <0.001 |
| High school | Reference | -17.409(-20.702,-14.116) | <0.001 | -22.112(-24.745,-19.478) | <0.001 | -28.280(-31.408,-25.152) | <0.001 | <0.001 |
| College or above | Reference | -14.505(-16.749,-12.260) | <0.001 | -19.956(-22.366,-17.546) | <0.001 | -23.766(-26.442,-21.089) | <0.001 | <0.001 |
| BMI (kg/m^2^) |  |  |  |  |  |  |  |  |
| < 25 | Reference | -19.131(-22.172,-16.090) | <0.001 | -24.652(-27.699,-21.606) | <0.001 | -27.951(-31.587,-24.315) | <0.001 | <0.001 |
| [25, 30) | Reference | -17.438(-20.429,-14.446) | <0.001 | -22.415(-25.109,-19.722) | <0.001 | -27.126(-30.096,-24.157) | <0.001 | <0.001 |
| ≥ 30 | Reference | -14.195(-15.889,-12.500) | <0.001 | -19.551(-21.844,-17.258) | <0.001 | -23.917(-26.473,-21.360) | <0.001 | <0.001 |
| Smokers |  |  |  |  |  |  |  |  |
| Never smoker | Reference | -17.803(-20.079,-15.527) | <0.001 | -22.137(-24.456,-19.817) | <0.001 | -27.431(-30.051,-24.812) | <0.001 | <0.001 |
| Former smoker | Reference | -14.923(-16.994,-12.851) | <0.001 | -24.612(-26.949,-22.275) | <0.001 | -27.718(-30.493,-24.942) | <0.001 | <0.001 |
| Current smoker | Reference | -13.275(-16.185,-10.364) | <0.001 | -17.164(-20.120,-14.207) | <0.001 | -21.235(-23.720,-18.751) | <0.001 | <0.001 |
| Alcohol drinkers |  |  |  |  |  |  |  |  |
| Nondrinker | Reference | -17.771(-19.908,-15.634) | <0.001 | -24.120(-26.170,-22.070) | <0.001 | -28.177(-31.085,-25.268) | <0.001 | <0.001 |
| Moderate alcohol use | Reference | -14.519(-16.991,-12.047) | <0.001 | -20.162(-22.199,-18.125) | <0.001 | -24.694(-27.206,-22.181) | <0.001 | <0.001 |
| High alcohol use | Reference | -11.814(-17.003, -6.624) | <0.001 | -14.912(-20.015, -9.809) | <0.001 | -18.966(-24.336,-13.596) | <0.001 | <0.001 |
| Hypertension |  |  |  |  |  |  |  |  |
| No | Reference | -13.885(-16.579,-11.190) | <0.001 | -17.094(-19.340,-14.847) | <0.001 | -19.895(-22.604,-17.187) | <0.001 | <0.001 |
| Yes | Reference | -13.140(-14.671,-11.609) | <0.001 | -18.298(-20.423,-16.173) | <0.001 | -24.502(-26.754,-22.251) | <0.001 | <0.001 |
| Cardiovascular diseases |  |  |  |  |  |  |  |  |
| No | Reference | -13.862(-15.459,-12.266) | <0.001 | -19.044(-20.739,-17.350) | <0.001 | -23.331(-25.480,-21.182) | <0.001 | <0.001 |
| Yes | Reference | -15.823(-18.890,-12.756) | <0.001 | -15.866(-19.653,-12.079) | <0.001 | -21.179(-25.990,-16.368) | <0.001 | <0.001 |
| Diabetes mellitus |  |  |  |  |  |  |  |  |
| No | Reference | -15.357(-17.176,-13.537) | <0.001 | -19.958(-21.783,-18.133) | <0.001 | -24.066(-26.281,-21.851) | <0.001 | <0.001 |
| Yes | Reference | -13.168(-16.093,-10.242) | <0.001 | -18.714(-22.190,-15.238) | <0.001 | -19.092(-23.949,-14.236) | <0.001 | <0.001 |

Notes: Q1 of moderate-to-vigorous physical activity (MVPA) was used as reference. Q1 (0-4.6 minutes/day), Q2 (4.6-14 minutes/day), Q3 (14-30.3 minutes/day), Q4 (30.3-313 minutes/day); Abbreviations: BMI, body mass index; ptrend, p for trend.
